# Tracing Neanderthal mobility through the Romanian Carpathians: A GIS-based least-cost connectivity model

**DOI:** 10.1371/journal.pone.0334149

**Published:** 2025-10-10

**Authors:** George Murătoreanu, Marian Cosac

**Affiliations:** 1 Department of Geography, Valahia University of Târgoviște, Târgoviște, Romania; 2 Department of History, Valahia University of Târgoviște, Târgoviște, Romania; Universita degli Studi di Ferrara, ITALY

## Abstract

Out of the 12,300 known caves within the Romanian Carpathian and Dobrudja mountain area, only 16 have been the focus of consistent archaeological researches which revealed traces of human activity (lithic artefacts, faunal material, combustion areas), roughly consistent with a Middle Palaeolithic technological and/or chronological background. Establishing natural areas connected in terms of accessibility to these caves may offer a different perspective for future researches and, above all, may increase the chances of discovering new caves with traces of human activity. The present study aims to integrate advanced spatial analysis methods, such as Linkage Mapper and Circuitscape, to assess the potential connectivity of these sites. The two models were developed by researchers in the field of biology and belong to the field of deterministic spatial modeling and algorithm-based geospatial analysis. Following the application of these models, we identified the areas of influence of the 16 caves, determined the least-cost paths between them and the main natural obstacles, in order to model spatial connectivity and identify new possible sites along these routes.

## 1. Introduction

The Carpathian Mountains represent one of the most complex and diverse geographical regions in Europe, characterized by a rugged mountainous terrain and a vast network of caves. These caves constituted shelters for prehistoric human communities, offering them refuge for shorter or longer periods of time [1,2]. In Romania, 12,300 caves are mentioned [3], most of them (8,637) being also included in an online database [4], in accordance with the systematic catalog of caves in Romania [5]. Of these, only a small part (around 100 caves) are in the extra-Carpathian space, namely in Dobrudja, the rest belonging to the Carpathian unit [4].

The use of caves by Neanderthal communities is one of the central themes in the study of the mobility and adaptability of these Paleolithic groups [6–10]. These caves were not just simple shelters, but functional points in a complex network of mobility and resource exploitation [6–14]. Despite the progress made in the research of Middle Paleolithic (hereafter, MP) sites in the Carpathians, the level of knowledge of spatial relationships and mobility corridors remains, in many respects, less developed than in Western European [15,16]. Therefore, the study of the connectivity of these caves and potential movement routes should become a priority direction in MP research in the karst area of Romania.

In this study, the concept of “connectivity” is treated in a functional and geographical sense, referring to the physical accessibility between archaeological sites, modeled on the basis of topography and proximity to resources. This approach aims to reconstruct short-term mobility and travel costs in the Paleolithic landscape. However, human connectivity can also include a demographic dimension, reflecting occupational continuity, intergenerational dispersion and cultural transmission. In this sense, there are models that provide a socio-spatial interpretation of the organization of Upper Paleolithic communities, based on settlement networks and population distribution [17]. In the same time, the “human existence potential” model [18] offers an integrated ecological approach, analyzing the habitability of regions according to climatic and geospatial variables, independent of actual mobility routes. Thus, although the present study focuses on the physical-geographic dimension of connectivity, integrating these demographic and ecological perspectives represents a valuable direction for future research, aimed at providing a holistic view of Paleolithic mobility.

GIS technologies and least-cost analysis tools, such as Linkage Mapper [19] and Circuitscape [20], have demonstrated their potential in diverse fields, from biodiversity conservation to the study of ecological networks [21–25]. These tools allow the generation of least-cost corridors and energy connectivity flows, using resistance-to-movement models and detailed topographic data. Despite this potential, their direct application to the study of Neanderthal mobility in the Carpathians is almost non-existent, which provides a fertile space for the development of new interpretative perspectives.

The main objective of this study is to reconstruct the mobility network of the Carpathian MP communities by integrating advanced GIS methods, with a focus on generating cumulative energy cost (CWD) rasters, identifying least-cost corridors (lin_corridors) and analyzing connectivity flows (cumcurmap). The aim is to identify optimal energy links between caves, highlight “hub” areas with high mobility intensity and assess the quality of the corridors based on the NLCC (Normalized Least Cost Corridors) score.

The working hypothesis starts from the premise that the karstic landscape and natural barriers have contributed to the modeling of preferential mobility routes, and the application of the Linkage Mapper and Circuitscape methods can realistically reproduce these routes. In this way, the present study not only fills a methodological gap in MP research in the Carpathians, but also opens comparative perspectives with the western regions of Europe, contributing to a better understanding of the adaptation strategies and territorial organization of human groups.

## 2. Materials and methods

### 2.1. Study area

**Fig 1:**
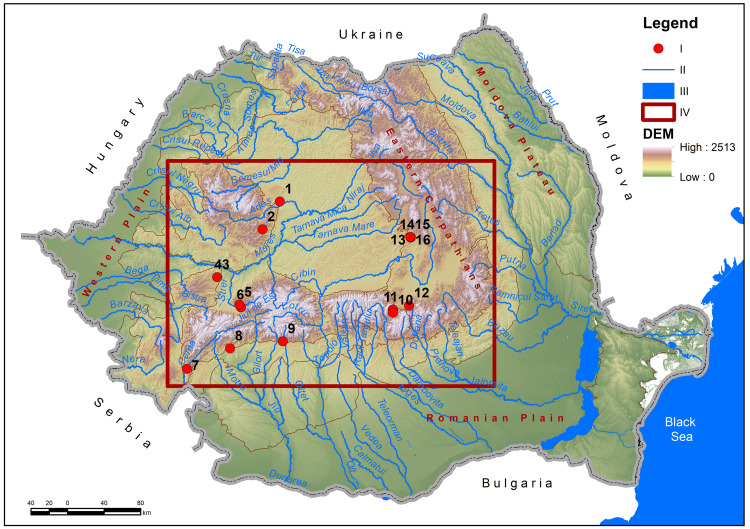
Location of the analyzed area in the Romanian Carpathian area: I. Archaeologically researched caves (1. Ungureasca Cave, 2. Peștereanu Cave, 3. Spurcată Cave, 4. Curată Cave, 5. Gaura Cocoșului Cave, 6. Bordul Mare Cave, 7. Hoților Cave, 8. Cioarei Cave, 9. Muierii Cave, 10. Sbârcioarei Cave, 11. Liliecilor Cave, 12. Gura Cheii Cave, 13. Abri 122, 14. Merești Cave, 15. Gabor Cave; 16. Liublinit Cave); II. Rivers; III. Lakes; Black Sea; IV. Limit of the analyzed area.

The area considered for the analysis is the space circumscribed by the 16 caves in the Romanian Carpathians (Fig 1) in which archaeological researches have been carried out (of which 6 have been identified and researched by the team coordinated by the authors of this study during the last 10 years). It is an area of 88185 km^2^, representing 36% of the entire surface of Romania. This overlaps in particular with the Carpathian area, centered on the Transylvanian Depression which occupies 21% of the entire area, surrounded by the Carpathians, with their three branches which together represent 52% of the entire analyzed territory. The rest of the territory overlaps with the Subcarpathians and the outer-Carpathian plateau and plain units.

The 16 caves analyzed are the only ones known so far in Romania to have sedimentary packages that include characteristic MP layers (chronologically and/or culturally defined) [11,12,26,27]. The sites analyzed in this study belong to the MP, being chronologically framed in a wide interval, corresponding to the MIS 6–3 climatic sub-stages (~130,000–40,000 BP). Although they all belong to the same technological and cultural framework, the occupations are not considered contemporaneous in the strict sense. Recent studies conducted in the region [1,2,28] have made significant contributions to the understanding of the chronology of the MP sites, demonstrating the existence of successive habitation events, in similar paleogeographic contexts. These studies confirm that certain caves were used in distinct but recurrent episodes, which indicates a sustainable and directed exploitation of the same landscape structures.

In this context, modeling minimum cost routes does not assume the synchronicity of occupations, but rather targets the potential for functional connectivity between important points for groups that shared the same geographical space in different eras of the Middle Paleolithic.

In relation to the main relief units, the caves with MP layers in Romania are as follows: 4 in the Western Carpathians (Ungureasca Cave, Peștereanu Cave, Spurcată Cave, Curată Cave), 8 in the Southern Carpathians (Gaura Cocoșului Cave, Bordul Mare Cave, Hoților Cave, Cioarei Cave, Muierii Cave, Sbârcioarei Cave, Liliecilor Cave, Gura Cheii Cave) and 4 caves in the Eastern Carpathians (Abri 122, Merești Cave, Gabor Cave, Liublinit Cave) (Table 1). It is important to add that there was no documentation of MP in the Eastern Carpathians prior to our researches. All caves with MP presences in the Eastern Carpathians were included in the specialized literature by the team led by the authors of this article [1,2,26,28].

**Table 1:** Location of caves with MP layers in Romania (X, Y coordonates in WGS 84).

Nr	Name	Absolute altitude (m)	Relative altitude (m)	X_coord. (East)	Y_coord. (North)	Relief Unit
**1**	Ungureasca Cave	590	110	23.68216117230	46.56276347700	Trascău Mts.
**2**	Pestereanu Cave	773	200	23.44315638440	46.28658374230	Metaliferi Mts.
**3**	Spurcată Cave	340	82	22.81529600680	45.80658848280	Poiana Ruscă Mts.
**4**	Curată Cave	320	86	22.81732501640	45.80642729000	Poiana Ruscă Mts.
**5**	Gaura Cocoșului Cave	756	280	23.13716331300	45.54214173080	Șureanu Mts.
**6**	Bordul Mare Cave	725	220	23.16057133690	45.51601803220	Șureanu Mts.
**7**	Hoților Cave	200	25	22.42951867650	44.89623757670	Cernei Mts.
**8**	Cioarei Cave	407	30	23.02376807240	45.10839941730	Vâlcan Mts.
**9**	Miuerii Cave	703	40	23.75634606080	45.18896517510	Parâg Mts.
**10**	Sbârcioarei Cave	914	25	25.29170337330	45.47836531840	Bran-Rucăr Corridor
**11**	Liliecilor Cave	1037	210	25.29343318300	45.50721253530	Bran-Rucăr Corridor
**12**	Gura Cheii Cave	782	6	25.51709904100	45.54643218800	Postăvaru Mts.
**13**	Abri 122	711	30	25.54617599980	46.21500900010	Vârghișului Mts.
**14**	Merești Cave	634	18	25.54614939200	46.21864716500	Vârghișului Mts.
**15**	Gabor Cave	688	77	25.54286500490	46.21963531550	Vârghișului Mts.
**16**	Liublinit Cave	695	72	25.54155545600	46.21991921350	Vârghișului Mts.

**Fig 2:**
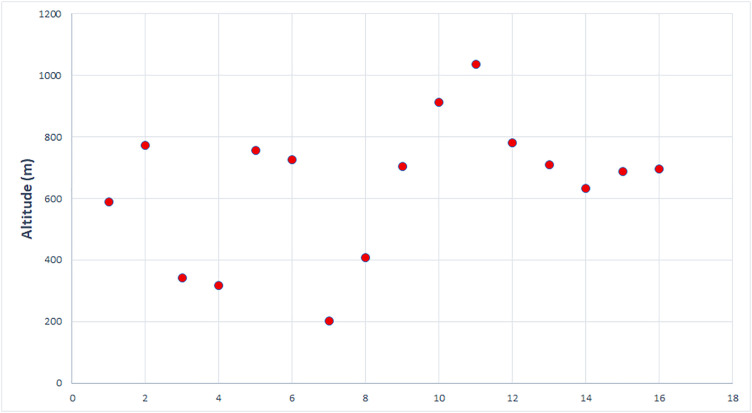
Altitudinal distribution of caves with MP layers.

Altitude-wise, most caves are in the 600–800 m range (Fig 2), with very high or very low altitudes being exceptions to this pattern. The only cave located below 300 m is Hoților Cave (Herculane), and the only one above 1000 m is the Liliecilor Cave (Bran-Rucăr Corridor), both in the Southern Carpathians.

The initial reporting and research of the MP in the Carpathian area is due to the archaeologist Márton Roska (1880–1961), as are the first chronological estimates, based on stratigraphic position, description of lithic material and faunal associations. From this stage onwards, all MP settlements were considered contemporary and belonging to the same cultural tradition [29], a point of view taken up in subsequent literature. The Roska stage, when the Gaura Cocoșului and Bordul Mare caves were investigated, was continued by C.S. Nicolăescu-Plopșor (1900–1968) and his students [30–32], with researches in caves such as Hoților, Curată, Spurcată, Boroșteni, Muierilor, Liliecilor, Gura Cheii [11,12].

After a period in which no new sites belonging to the MP were highlighted, recent archaeological researches were carried out in the caves Abri 122, Merești, Gabor, Liublinit (Vârghiș Gorges – Perșani Mountains) [26] and Peștereanu (Apuseni Mountains) (unpublished information).

In previously published works, the analyzed caves were considered to gather a series of criteria that met the requirements of human communities: orientation, dimensions, absence of air currents, proximity of a depression and of possible observation points as part of a hunting strategy, proximity of water resources and sources of raw materials [11,12,33]. These criteria seemed valid until recently, since the caves in Vârghiș Gorges, as well as Peștereanu Cave do not fit into this pattern [1,2,26].

The same situation is also recorded regarding the absolute chronology of the MP assemblages in these caves, requiring a chronological reassessment of older excavations [14]. The recently researched sites in Vârghiș Gorges indicate a MIS 3 – MIS 4 chronology [28].

### 2.2. Input data

The input data for this study were obtained from several geospatial data sources and from field work. The exact location of the caves was obtained using GPS instruments directly from the cave portal area, where we benefited from our own investigations [1,2,28,34,35]. For the caves whose research did not belong to us [11–14] the location was done using bibliographic references, their identification in the field and their verification using Google Earth.

For the relief analysis, we used the Digital Terrain Model resulting from the SRTM mission (Shuttle Radar Topography Mission) available, at a resolution of one arc second (about 21x30 m), from the USGS earthexplorer website [36].

The hydrographic network was cut from the Romanian hydrographic network in vector format provided by the Copernicus portal, part of the European Union Space Program. It provides information derived from data provided by the European Space Agency’s Sentinel satellites, as well as data obtained from terrestrial networks. The program has six thematic services: Atmosphere Monitoring, Marine Monitoring, Land Monitoring, Climate Change, Security and Emergency Management. The Romanian hydrographic network is part of the Danube Basin dataset [37].

This database includes both major, permanent streams and some secondary arteries but of sufficient size to be included in a European database. No differential weights or selective calibrations were applied to adjust for the influence of water access, as all three criteria used in constructing the cost raster—altitude, slope and distance from water—were weighted equally, in order to maintain a balance between accessibility factors.

Although the exact position of the Middle Paleolithic hydrographic arteries cannot be known with precision, it is considered that, in the mountainous and hilly regions connected to the Carpathian arc, the major valley network was relatively stable during the Quaternary. The main evolutionary trend was the deepening of pre-existing valleys, not their lateral migration or the creation of completely new routes. This stability is supported by geomorphological studies applied to Romanian river basins, which indicate dominant processes of fluvial incision and terrace formation [38–41]. In this context, we consider that the use of the current hydrographic network as a proxy for paleohydrography is justifiable for regional modeling.

### 2.3. Methodology

To analyze the spatial relationships between caves considered to be occupied in the same chronological and climatic stages [1,11,12,14,28,33] we used two GIS protocols developed in the biological world, for two reasons: the movements of Neanderthal communities could be connected to the movements of herds of herbivorous mammals and their behaviors could be likened to those of large predators [42]. Neanderthal communities’ movements can be correlated with seasonal movements of herbivore herds, especially in paleogeographic contexts where the migration of large prey (reindeer, horses, cattle) was predictable. In such a framework, Neanderthal mobility seems to have been structured according to the distribution and availability of resources, with variable but repetitive adaptive strategies [43,44]. A recent study [45] demonstrates that decisions regarding the transport of carcasses were made according to the effort of movement and caloric value, suggesting a rational and cost-efficient behavior. Thus, even if Neanderthals do not fit the ecological typology of large predators, their spatial behavior can be partially understood through functional analogies—especially in terms of responding to the dynamics of mobile resources. This logic is compatible with the application of circuit theory [25], which models spatial connectivity in contexts where individuals have limited but directed knowledge of the landscape. This theoretical framework has already been used in paleoanthropological studies to model the migration of early humans from Africa, using Circuitscape, taking into account terrain resistance and natural barriers [46] or to demonstrate that the complex relief of the Caucasus affects human dispersal and gene flow [47]. Therefore, the application of this theory to Neanderthals in the Carpathian space is not only methodologically justifiable, but also empirically supported by similar studies.

The protocols used were Linkage mapper [19] and Circuitscape [20]. These are two GIS applications used especially in landscape ecology and the identification and conservation of habitat connectivity, and we ran them using the ArcGIS 10.6 package.

Linkage Mapper was developed to identify and model ecological corridors between priority habitat areas, facilitating the conservation of functional connectivity for wildlife species. This tool is based on the modeling of least-cost paths between habitat “cores”, providing an intuitive and accessible approach to connectivity planning [23]. Circuitscape, on the other hand, applies the theory of electrical circuits to simulate species movement and gene flow through fragmented landscapes. This program proposes a different way to understand ecological corridor analysis, replacing single least-cost routes with an approach that considers multiple possible paths, similar to electric current in a network [22]. Today, both tools are used at scale in ecological network planning, in green infrastructure projects, as well as in climate change adaptation strategies. Linkage Mapper is preferred in regional-scale planning contexts due to its simplicity, while Circuitscape is used in complex analyses, such as those involving genetic connectivity or species movement scenarios under variable habitat conditions [25]. With the help of these packages, projects have been developed in Romania for the identification and protection of ecological corridors for large carnivores in the Carpathians or for the management of transboundary ecological corridors by WWF Romania [48].

In identifying possible routes of Neanderthal communities, we started by processing the three parameters considered important for conditioning movement (altitude, slope and proximity to water) and including them in a movement cost raster (Table 2). Least-cost paths were calculated based on this energy cost raster, in which slope was calculated from the DEM, absolute elevation, also extracted from the DEM, was penalized at over 1200 m, and distance from water, created based on a buffer, was penalized at distances greater than 2 km. These variables were combined into a final raster expressed in relative cost units, without an absolute physical unit, but relevant for comparing the optimal route between sites.

**Table 2:** Scores given to the environmental elements considered.

Points	Altitude	Slope	River distance
**1**	300 - 1200	0–10	< 1 km
**3**	<300	10–20	1–2 km
**6**	1200 - 1500	20–30	2–4 km
**10**	> 1500	> 30	> 4 km

The three variables were given increasing values in relation to the difficulty of crossing the landscape under the conditions imposed by their characteristics. The points awarded were 1, 3, 6 and 10 to clearly differentiate the easily accessible areas from the more difficult ones. The exponential increase in the values reflects a similar exponential increase in energy costs as the terrain becomes more resistant, being a more efficient variant in relation to the linear increase in the values which underestimate the real increase in resistance on difficult terrain [49].

The altitude, derived from the DEM, was classified based on the idea that the most accessible space is between 300 and 1200 m, a space that corresponds to the level of hills and low mountains, the most attractive from a landscape point of view [7,8], with numerous shelter possibilities, observation points and fluvial terraces characterized by low slopes. Paleoecological analyses have shown that numerous MP sites are located in low to medium levels, indicating behavioral adaptations in accordance with the climatic and energetic advantages of these altitudes [50]. This space was given the value 1, being the most offering space for movement. In fact, with one exception – Hoților Cave, all the other caves under discussion are located in this hypsometric interval. The next class is the hypsometric step below 300 m, corresponding to the current plains. Not having much evidence of Neanderthal activity here, this space was given the value 3. The other classes (6 and 10 points) were assigned to the hypsometric steps 1200–1500 and above 1500 m, considering that hunter-gatherer populations avoided these inhospitable altitudes even at the level of the last interglacial.

The distance from water was achieved starting from a multiple buffer applied to the current hydrographic network, with steps ranging between 1, 2, 4 and over 4 km. These steps were assigned the same values.

**Fig 3:**
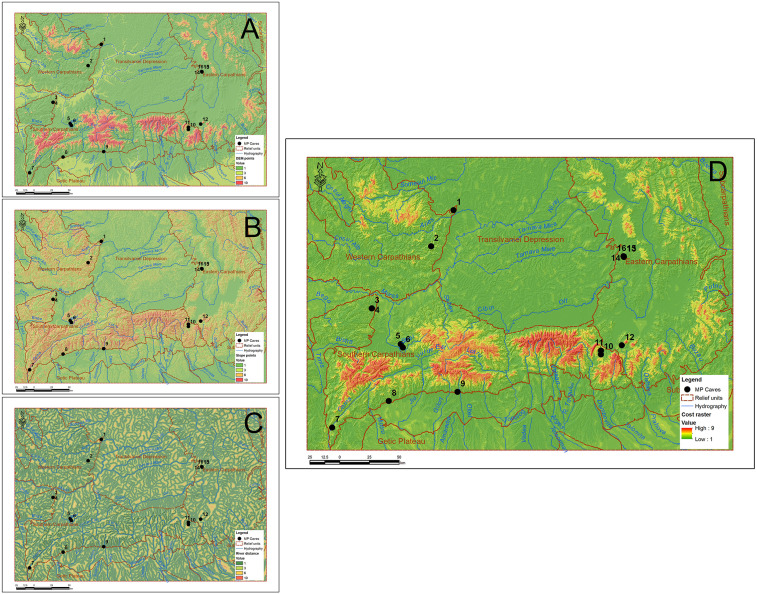
Modeling of the cost_raster grid (D) based on the scores given to hypsometry (A), slopes (B) and distance from the hydrographic network (C).

From the three rasters with the values thus calculated, using the raster calculator function, the cost raster was created, which formed the basis of the analysis using Linkage Mapper and Circuitscape (Fig 3).

The next step was to transform the point vectors corresponding to the MP sites into polygon vectors corresponding to the core areas, vectors accepted by the Linkage Mapper and Circuitscape protocols. The dimensions of the polygons were equal because we cannot make a hierarchy of the dimensions or intensity of the dwellings in the mentioned caves.

Starting from the cost raster (cost_rast) and the core areas (core_areas), the two complementary protocols that are the subject of this article were applied:

Least-cost modeling (Linkage Mapper) was performed to identify direct, energetic corridors between the sites. Linkage Mapper is an open-source GIS tool designed to identify spatial connectivity corridors between core areas, based on a cost surface raster. It generates connecting paths that minimize the energetic cost, not the geometric distance. The model is based on the Least-Cost Path (LCP) theory and computes a network of corridors between all possible pairs of source sites. The costs are accumulated in the cumulative weighted distance (cwd) raster, and then alternative routes between connectable cores are compared.

The term “energy” was used as an abstract unit expressing the cumulative spatial cost of travel. This is derived from a rasterized cost model, in which each cell was weighted by local slope and relative elevation, also taking into account distance from water. The model does not directly quantify metabolic energy (e.g., calories burned) or travel time, but rather provides a functional proxy for the overall difficulty of traversing the terrain. The resulting cumulative cost surfaces thus express a relative impedance, with lower values signaling more energy-efficient routes. For interpretation, we divided these values into three ranges:

- 0–100 units – direct routes, with minimal costs (interpreted as likely and repetitive routes);
- 100–5,000 – secondary corridors, possibly seasonal or logistical;
- 5,000–10,000 – peripheral routes, with possible frequency but high costs, reflecting geographical constraints.

These thresholds were established heuristically, by visual correlation with the hydrographic network and physical distances between sites, and can be adjusted for future sensitivity analyses. Probabilistic modeling (Circuitscape) was used to highlight emergent routes, based on electrical flow. Circuitscape applies the theory of electrical circuits to spatial ecology and, in our case, to modeling the possible movements of MP groups. Unlike the LCP model, Circuitscape does not compute just a single optimal route, but all possible routes between points, distributing “current” through the resistance raster (equivalent to cost_raster). Thus, a probabilistic picture of mobility is obtained.

To generate the spatial graph in Circuitscape, the raster cells were connected in an 8-neighbor configuration (a “queen’s case” connection), meaning that each cell is connected to both its orthogonal and diagonal neighbors. This setup provides a much more realistic simulation of mobility in complex landscapes—a central principle in applying circuit theory, which assumes the existence of multiple alternative paths between nodes. The Circuitscape user guide explicitly describes this option as using either four first-order or eight first- and second-order neighbors [22].

## 3. Results and discussions

Following the application of the two protocols, data and graphic materials resulted that may change the perception of the mobility of Neanderthal communities in the Romanian Carpathians.

### 3.1. Areas of influence and direct connectivity

The zones of influence of archaeological sites were defined as the areas around each site where access is easiest (with the lowest energy cost) compared to other sites in the network. In other words, each zone indicates the territory where a site is most functionally accessible, based on the applied mobility model. These zones help to understand the relative spatial role of each site and to assess the potential connectivity between them.

**Fig 4:**
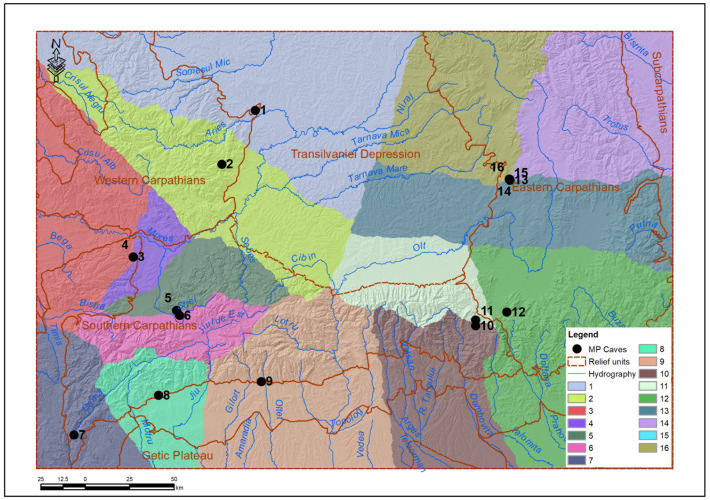
The cwd_alloc_ras raster resulting from the application of the Linkage Mapper protocol highlights the energetically allocated spaces for each cave (1–16: caves numbered according to Table 1).

**Fig 5:**
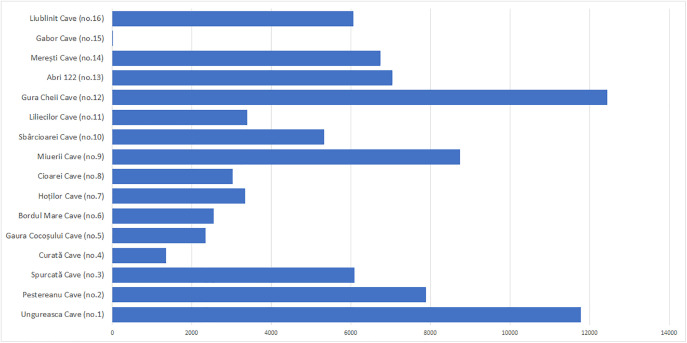
Areas allocated to each cave, according to the cwd_alloc-ras raster.

According to Linkage Mapper, the first stage provided an influence map - cwd_alloc_ras, which signifies territories of influence based on cost (Fig 4), delimiting the areas in which each core (cave) exerts an energetic dominance. These areas, delimited by cumulative cost, highlighted how topography and natural resources determine the isolation or overlap of nuclear territories. The cwd_alloc_ras raster reflects the areas of influence of each Neanderthal site, defined not by Euclidean distance, but by energetic distance (depending on relief, slope and proximity of water). It shows for each pixel on the map which site would have been the most accessible in terms of movement effort, thus achieving the natural delimitation of “functional territories” assigned to each cave. According to this raster and the resulting allocated area graph (Fig 5), Gura Cheii Cave (no. 11) has one of the most extensive areas of influence, being well positioned between depressional corridors, with easy access. Caves such as Peștereanu (no. 2) and Ungurească (no. 1) also dominate large areas due to their peripheral, but topographically favorable position. In contrast, the caves in the Vârghiș Gorges (no. 13–16) are very close to each other, forming a “cluster”. Spatial competition leads to a fragmented and extremely restricted allocation, especially for Gabor Cave, which has an allocated area of only one km^2^.

In the context of this study, the notion of “natural resource” refers mainly to surface water sources, karst shelters (caves and limestone cliffs) and, interpretatively, to the abundance of fauna that could be hunted. Of these elements, only distance from water was directly integrated into the cost raster, being considered a functional constraint on mobility. Data on the presence of lithic resources or fauna, although archaeologically relevant, could not be explicitly included in the model due to the lack of paleoclimatic and paleoecological spatial layers with adequate resolution. These are mentioned in the contextual interpretation as potential factors in movement decisions.

The territorial distribution is uneven, with clear centers of mobility and areas of energetic periphery. Some caves function as cores of regional influence – e.g., Gura Cheii dominates the entire depression of Țara Bârsei. Others are almost nullified as an influence, not being able to “win” any space in the energetic competition (e.g., Cave 15). The model reveals that the density of sites in a region can lead to spatial over-crowding, which drastically reduces the individual efficiency of a core.

**Fig 6:**
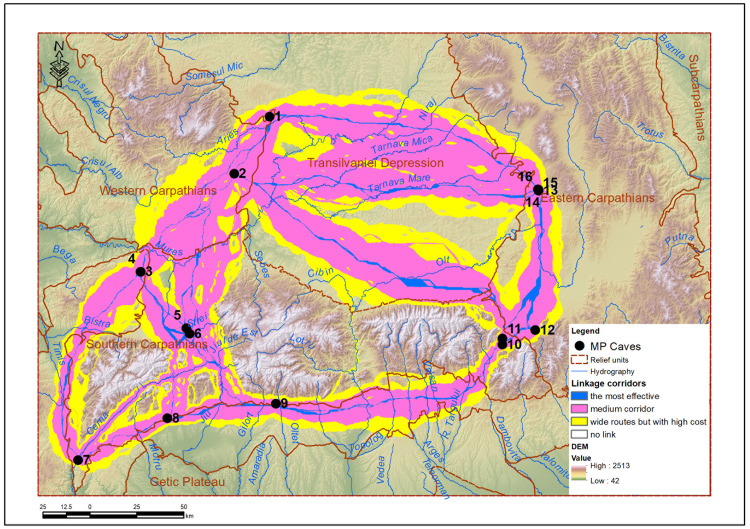
Main corridors between MP sites in the Romanian Carpathians.

The connectivity corridor map – the lin_corridors raster – is the main product of the Linkage Mapper protocol. It represents functional connectivity corridors between pairs of sites, not as single lines, but as spatial bands with varying strengths. To increase readability and focus only on the most significant routes, the lin_corridors raster was limited to a threshold value, set at 10,000. This value represents an arbitrary, but empirically justifiable limit, which highlights the most used corridors. The resulting map (lin_corridors_truncated_at_10k) provides a clear picture of the potential mobility axes predominantly used by the MP communities (Fig 6). The lin_corridors_truncated_at_10k raster reflects the density of least-cost paths (LCP) between the analyzed sites, each pixel accumulating values depending on the frequency with which it is crossed by these routes. This approach allows the identification of the most likely corridors of Paleolithic mobility, highlighting coherent patterns of spatial connectivity. For efficient interpretation, the values were classified into three meaningful ranges, reflecting the energetic efficiency of the routes:

The range 0–100 (blue) highlights primary mobility corridors, characterized by the lowest energy cost. These represent the most efficient and, most likely, the most frequented routes between sites, suggesting direct and recurrent routes in the logic of Neanderthal mobility [22,51]..

The range 100–5,000 (pink) includes secondary routes, slightly more expensive but still accessible. They may reflect seasonal variations, alternative logistical options, or occasional extensions of the main routes.

The range 5,000–10,000 (yellow) indicates areas with high frequency of crossing, despite a higher energy cost. These may correspond to detour routes, used in contexts of restrictive terrain, limited access, or dispersed resources. Although less efficient, they complete the functional mobility network, providing adaptive routes in the face of environmental constraints.

Values higher than 10,000 have been removed to avoid visual overload and to exclude regions with prohibitive energy costs, such as high mountain ridges (e.g., above 1,500 m). This threshold is ecologically and methodologically justified [52], by applying filters to maintain the interpretative relevance of the corridors. The application of the 10,000 threshold is specific to the current analysis, as it was found that above this threshold the values covered the entire map, losing their discriminative significance. Eliminating these values allows for a focus on functional, realistically accessible routes, in accordance with the principles of least-cost modeling in spatial ecology and prehistoric human mobility.

Running Linkage Mapper with the “pairwise” option (each with each), the program should have calculated all possible combinations between sites. Starting from 16 caves, there should have been 120 unique combinations (16 × 15/ 2). However, the protocol restricted the calculation of combinations to 40 links between caves, which if considered round-trip become 80 (Table 3), excluding those routes that are energetically impossible (either the energy cost is too high, or the caves are too far away and are separated by large obstacles). This automatic filter reflects a plausible ecological and ethological reality: Neanderthal groups would not have invested a disproportionate effort to travel on dangerous or very high energy consumption routes. Thus, the final set of corridors generated by Linkage Mapper – and exported in the form of rasters and the lin_corridors_truncated vector – can be considered a real functional mobility network, adapted to the natural limitations of the karst and mountain environment of the Carpathians.

**Table 3:** Number of links generated for each cave and the caves they are connected to.

Nr	Name	Number of links	The connected caves
**1**	Ungureasca Cave	4	2, 3, 13, 16
**2**	Pestereanu Cave	9	1, 3, 4, 5, 6, 9, 11, 13, 16
**3**	Spurcată Cave	7	1, 2, 4, 5, 6, 7, 8
**4**	Curată Cave	4	2, 3, 5, 6
**5**	Gaura Cocoșului Cave	6	2, 3, 4, 6, 8, 9
**6**	Bordul Mare Cave	7	2, 3, 4, 5, 7, 8, 9
**7**	Hoților Cave	3	3, 6, 8
**8**	Cioarei Cave	5	3, 5, 6, 7, 9
**9**	Miuerii Cave	6	2, 5, 6, 8, 10, 11
**10**	Sbârcioarei Cave	3	9, 11, 12
**11**	Liliecilor Cave	6	2, 9, 10, 12, 13, 16
**12**	Gura Cheii Cave	3	10, 11, 13
**13**	Abri 122	6	1, 2, 11, 12, 14, 16
**14**	Merești Cave	3	13, 15, 16
**15**	Gabor Cave	2	14, 16
**16**	Liublinit Cave	6	1, 2, 11, 13, 14, 15

Movements between sites should not be understood as actual routes, but as potential accessibility networks, reflecting the distribution of resources and the natural structure of the terrain. Studies show that mobility at the MP level was often strategically directed: not all shelters served as residences, some functioned as logistical points or as landmarks within seasonal exploitation [53,54]. Therefore, even long, seemingly “unrealistic” routes may indicate actions motivated by ecological constraints (lack of local sources of flint, need for access to hunting or water). Thus, least-cost modeling becomes a heuristic, not predictive, tool to understand the spatial potential of movement and not necessarily the actual mobility.

From the list of possible combinations (Table 3) some conclusions can be drawn: Corridors with very high energy costs were excluded. Routes between caves that are close or on the same access corridors (wide valleys, karst corridors) were preserved. The average number of connections for a cave is 5, varying between 2 and 9 connections. Most caves have 3–6 direct connections – suggesting a robust mobility network, but realistically limited by the geography of the region. The Peștereanu, Spurcată and Bordul Mare caves, with 9 and 7 connections respectively, can be considered important connectivity nodes – potential hubs of the Neanderthal network. This concentration of connections reflects the real possibilities of movement and the potential for access to resources and adjacent territories.

**Fig 7:**
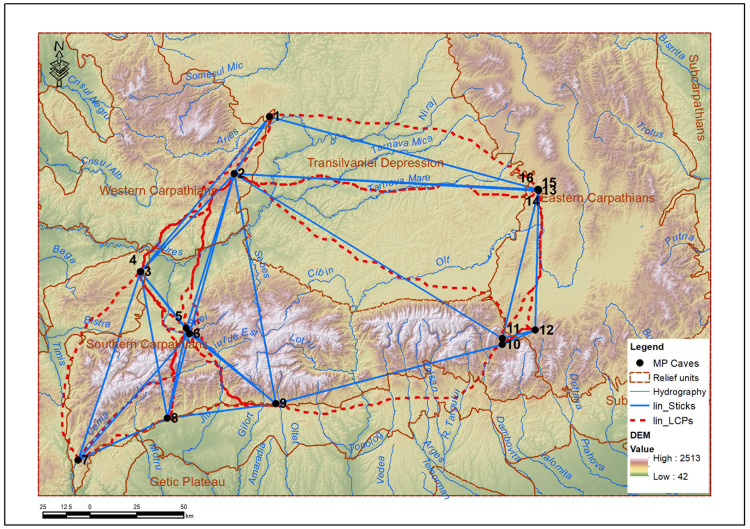
The Least-Cost Paths (lin_LCPs) and the straight-line (lin_Sticks) distances between the analyzed pairs of sites.

A key analysis in validating the models is the comparison between the modeled connectivity and the intensity of documented archaeological evidence at the sites. For example, Peștereanu Cave, located in the Apuseni region, has one of the largest areas of influence and the highest number of connections (9), which corresponds to the large volume of artifacts and the complex stratigraphy observed in recent research. Similarly, Curată Cave and Bordul Mare Cave are well connected in the network and correspond to sites with rich stratigraphic occupation. In contrast, Gabor Cave, in Cheile Vârghișului, has an extremely small area of influence (only 1 km²) and appears poorly connected, which may suggest a punctual or specialized use. Other sites, such as Hoților or Ungureasca, although frequently mentioned in classical literature, appear marginal in terms of connectivity, which may reflect either restrictive geographical conditions or seasonal or chronologically limited use. These discrepancies indicate the need to re-evaluate some sites using modern methods, but also the potential of these models to suggest places that require extensively archaeological investigations. For the |energy-efficient routes, Linkage Mapper generated the vector lin_LCPs (Least-Cost Paths) which represents the most energy-efficient routes (with the lowest accumulated cost) between each pair of sites (core areas), calculated based on the cost raster. At the same time, for the same pairs of sites, it also generated the straight-line distances (not the cost-optimized ones) between the analyzed pairs of sites – lin_Sticks (Fig 7). By relating the straight-line distances to the real distances, we can identify the most difficult routes and the simplest ones. In our case, the easiest link (a ratio of 1.02) is between Cioarei Cave and Muierii Cave (real path: 59,9 km and straight-line distance: 58,2 km) and the most complex link (with a ratio of 1.38) is between Peștereanu Cave and Muierii Cave (real path: 171,7 km and straight-line distance: 124,3 km).

### 3.2. Energy flow and probabilistic mobility

Circuitscape modeling extended the analysis by including all possible paths between sources, providing a probabilistic perspective on mobility. Circuitscape uses an analogy with electric current to simulate how flow “flows” between sources through a resistive medium [22,23], in our case represented by the cost raster in Fig 3, the same one used for Linkage Mapper. In an archaeological context, this translates into identifying areas where movement between sites is more likely, due to lower transit costs. Unlike classical least-cost path models, Circuitscape does not calculate just one optimal route, but considers all possible routes, generating a distributed flow network.

This protocol allowed the generation of several types of raster maps, each providing a complementary perspective on human groups mobility in the mountainous karstic landscape.

The cwd (cumulated weighted distance) rasters, resulting from the Weighted Accumulated Cost calculation for each of the 16 source sites, express the cumulative difficulty of reaching from each site to the entire space around it, taking into account the specific resistance of each cell in relation to slope, altitude and distance from water. The values obtained, ranging from 0 to over 400,000 (cumulated weighted kilometers) cw-km, were limited to a threshold of 100,000 cw-km, considering that beyond this threshold accessibility is no longer directly influenced by landscape characteristics, distance being the one that limits access. The cw-km unit expresses the cumulative distance weighted according to the resistance of the environment (slopes, altitude, obstacles), not the real physical distance. Thus, 1 km traveled through difficult terrain can be equivalent to tens of cw-km. This metric is frequently used in least-cost models to reflect the real travel effort. The 100,000 threshold is similar to those tested in natural habitats in North America [49,55] and exclusion thresholds of 100,000–120,000 cw-km were used, which are considered feasible connectivity limits and are also applied in this study to eliminate energetically improbable corridors.

**Fig 8:**
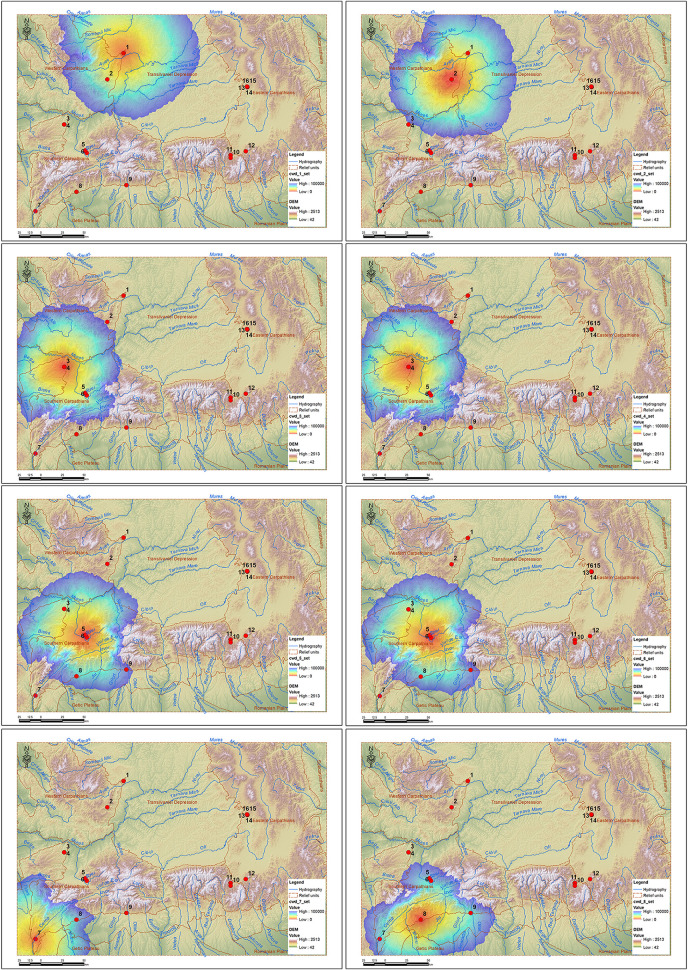
CWD rasters for sites 1-8 (1. Ungurească Cave; 2. Peștereanu Cave; 3. Spurcată Cave; 4. Curată Cave; 5. Gaura Cocoșului Cave; 6. Bordul Mare Cave; 7. Hoților Cave; 8. Cioarei Cave).

**Fig 9:**
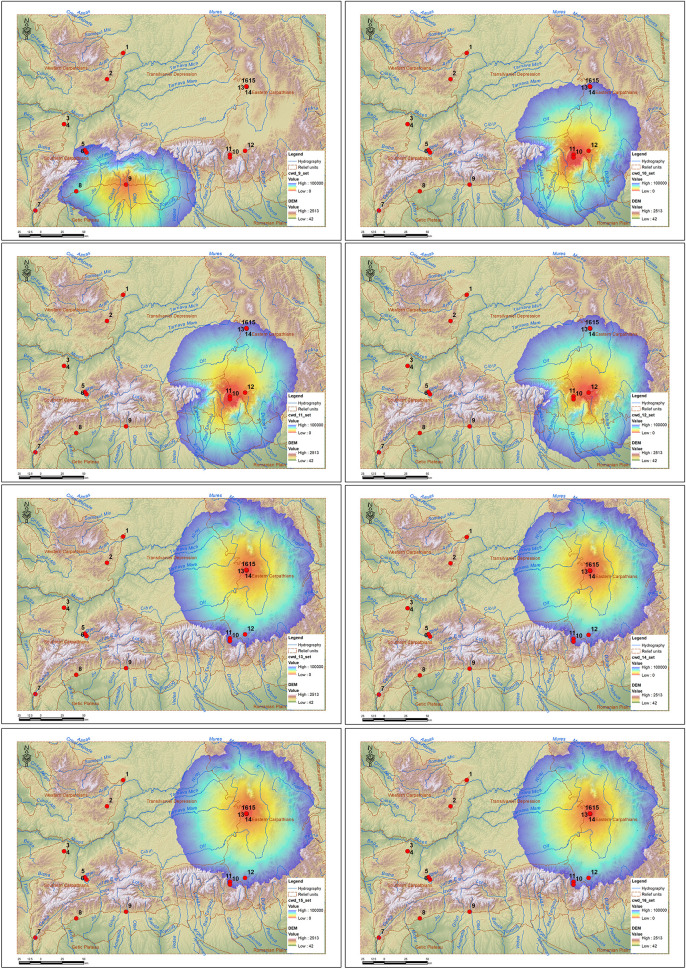
CWS rasters for sites 9-16 (9. Muierii Cave; 10. Sbârcioarei Cave; 11. Liliecilor Cave; 12. Gura Cheii Cave; 13. Abri 122; 14. Merești Cave; 15. Gabor Cave; 16. Liublinit Cave).

Depending on the relief characteristics, this threshold is equivalent to a distance between 25 and 70 km around the sites, which is in full agreement with the specialized literature. Flint provenance analyses indicate a regional mobility pattern of human communities, with frequent movements in territories of about 50–60 km in diameter, depending on lithic sources and landscape structure [7]. This supports the use of the aforementioned threshold for delimiting energetically viable corridors, excluding areas with excessive cost, improbable for Paleolithic human movement. Interpretation of these maps (Figs 8 and 9) shows that sites located in mountain depressions and corridors generate extensive and homogeneous areas of accessibility, while sites located at high altitude present areas of reduced influence.

In the case of caves surrounded by an intense red area (low cost values), it indicates an easily accessible area, which radiates influence uniformly around, as in the case of the Peștereanu Cave (Fig 8), Curată and Spurcată Caves (Fig 8), Bordul Mare (Fig 8) or the caves in the Vârghiș Gorges (Fig 9).

Areas with obvious asymmetries show natural relief barriers, which direct or block accessibility. This is the case of Cioarei Cave and Muierii Cave blocked to the north by the Vâlcan and Parâng Mountains (Fig 8), or the caves in the Rucăr-Bran Corridor (Liliecilor and Sbârcioarei) with non-uniform areas (Fig 9) due to the presence of the Făgăraș Mountains to the west, the highest mountains in the Romanian Carpathians. Areas that show a rapid discoloration towards blue indicate relatively isolated caves, with limited territorial influence, such as the Ungureasca Cave, or Hoților Cave (Fig 8).

Areas with a wide red-yellow gradient may correspond to favorable mobility networks. Caves in intra-mountain depressions (Peștereanu Cave, Spurcată Cave, Curată Cave, Gaura Cocoșului Cave, Bordul Mare Cave) have increased accessibility (Fig 8). Caves located at the edge of slopes or in high mountainous areas (Hoților Cave – Fig 8, the caves from the Vârghiș Gorges – Fig 9) are more difficult to reach energetically, with high travel costs.

For each site, a voltage map was also calculated, which expresses the potential for electrical propagation in a resilient landscape. The voltage map rasters, resulting from running the Circuitscape algorithm, measure the importance of each site as a node in the connectivity network, based on an analogy with electrical flow: sites that concentrate high voltage values contribute more significantly to the connection of the entire network. Thus, these maps allow the identification of key points whose loss would severely affect potential mobility, indicating a strategic value for the preservation of connectivity or for understanding the spatial behavior of Paleolithic communities.

Based on them and on the cwd rasters, the cumcurmap raster was generated.

**Fig 10:**
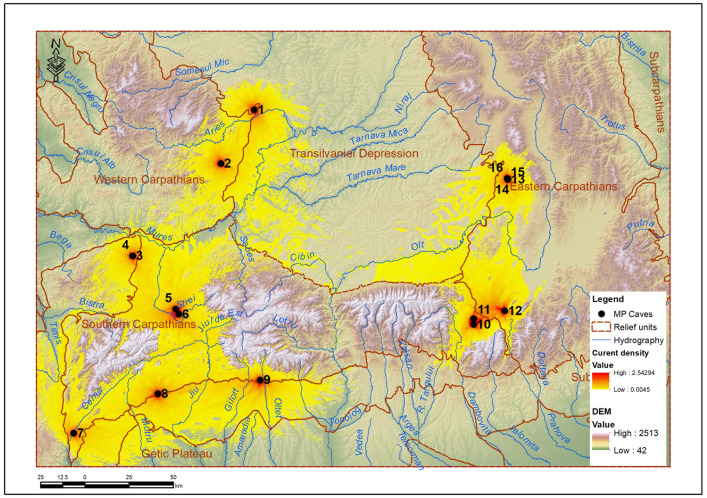
Cumulative flow map (cumcurmap).

The cumcurmap raster – cumulative flow map (Fig 10) – defines the cumulative flow and emerging routes, starting from the 16 cwd (cumulated weighted distance) rasters. The cumcurmap raster highlighted the areas with the highest “current” flow – that is, emerging mobility routes, validated not only as optimal routes, but as a result of topography and contextual connectivity. Cumulating the calculated current between all pairs of sites produced a general flow map (cumcurmap) that highlights the preferential mobility routes. This map provides an emerging model of global connectivity between sites, in which energy density reflects both distance and ecological conditions of the possible routes.

In the map obtained for the MP sites in the Romanian Carpathians, high flux values (e.g., > 0.02) are concentrated around natural passage routes: the Bran-Rucăr Corridor, the Hațeg Depression, the Jiu Valley and to a lesser extent the Olt Valley. These “convergence” areas correspond to strategic locations that would have allowed the efficient connection between the intra-Carpathian basin and the exterior of the Carpathian arc. For example, between the sites in the Apuseni Mountains (Ungurească Cave, Peștereanu Cave) and those in the Getic Subcarpathians (Muierilor Cave), the major flux is channeled through the Mureș corridor and the Jiu Valley. In the Eastern Carpathians, the Caves from the Vârghiș Gorges are connected by high flow with those of the Brașov Depression and the Bran-Rucăr Corridor through the Olt Valley and its tributaries (Bârsei Valley, Moieciu Valley, Sbârcioarei Valley).

The map synthesizes the energetic convergence between all possible routes and allows the identification of essential mobility nodes (Brașov Depression, Hațeg Depression, Mureș – Arieș Corridor, Bran-Rucăr Corridor and the contact between the Southern Carpathians and the Getic Subcarpathians). This representation complements the results from Linkage Mapper and cwd, providing an integrative picture of the real probability of movement, validating the modeled network as coherent and logical from a geographical and energetic point of view.

### 3.3. Comparison of results and convergent areas

Linkage Mapper provides clear but unique routes between sites. Circuitscape, on the other hand, provides a continuous and probabilistic picture of the overall flow. While Linkage Mapper helps us identify preferred axes, Circuitscape suggests the distribution of mobility pressure across the landscape.

The comparison of the two methods highlighted a series of mutually validated corridors (for example, on the corridors between the Apuseni and Subcarpathian Mountains or the depressional areas of the Eastern Carpathians), confirming the routes interpreted as preferential in Neanderthal mobility.

The integrated analysis indicates a series of stable corridors (Brașov Depression, Hațeg Depression, Mureș – Arieș Corridor) that appear in both models. These can be interpreted as recurrent routes, probably used over millennia by several communities.

The sites with the highest number of connections (resulting from the analysis of the lin_corridors and res_voltmap rasters) were identified as regional nodes – for example, the caves in the Varghis Gorge or in the Apuseni Mountains. For example, Peștereanu Cave seems to be of major importance in terms of connectivity and position in the ensemble of caves in the Carpathians where MP was documented. In contrast, other sites, although archaeologically important, appear isolated from the perspective of spatial connectivity.

Based on the cwd and lin_corridors rasters, caves with high centrality were identified (such as Peștereanu Cave, Curată Cave or Bordul Mare Cave), which appear connected to several sites. Other caves, such as those in the Vârghiș Gorges, appear relatively isolated.

### 3.4. Discussions

A major limitation of the model is the use of modern topographic and hydrographic surfaces as a proxy for the landscape 40,000–60,000 years ago. Although the overall morphological structure (elevation, slope, karst relief arrangement) has remained largely stable since the end of the Middle Pleistocene, river courses, alluvial deposits and the degree of accessibility to karst inlets may have been significantly modified by postglacial processes.

Thus, it is possible that some low-cost corridors were in reality temporarily inaccessible, or that routes modeled as unlikely were favored in the paleolandscape.

Therefore, the models presented should be understood as relatively plausible functional scenarios, which provide a comparative framework for analysis, but do not reflect the absolute fidelity of the Paleolithic landscape.

Some of the modeling results also seem to be supported by indirect signals from archaeological contexts. For example, at the Mare and Hoților sites, connected by low-energy corridors, lithic raw materials of different provenance were identified, as well as indications of repeated use, such as successive hearths and processed fauna. This evidence may reflect recurrent regional mobility between shelters, which supports the functional realism of the modeled routes.

Although we did not implement a leave-one-out test, the behavior of the network suggests that most real sites are located in the proximity of high-connectivity corridors, generally at distances below 500 m. Thus, the model has relevant prospective potential, by delimiting target areas that can guide future archaeological investigations.

Areas where multiple modeled routes overlap can be interpreted as functional nodes of the MP network—spaces where occupations, shelters, or transition points are likely to have existed.

In an applied setting, it is recommended to explore the terrain in a buffer of 500–750 m along low-cost corridors, especially in poorly researched karst regions. A systematic leave-one-out test may constitute a future step to formally validate the predictive accuracy.

## 4. Conclusions

This study demonstrates the relevance of the integrated use of Linkage Mapper and Circuitscape in the connectivity analysis of MP sites in the Romanian Carpathians. The results showed that the mountain landscape generated preferential mobility routes, and the connectivity corridors highlighted by both models converge in key regions such as the Hațeg Depression, the Mureș-Arieș Corridor, the Bran-Rucăr Corridor or the Brașov Depression. The obtained corridors allow the identification of spaces with a high probability of use during the course of the MP and provide a coherent basis for future archaeological explorations.

The cost raster was built starting from essential geographical variables—altitude, slope and distance from water sources—and allowed the realistic simulation of natural barriers and movement facilities. The model was calibrated using non-linear increasing cost values (1–3–6–10), reflecting the increased difficulty in crossing rugged areas, based on the specialized literature.

Through Linkage Mapper, least-cost corridors between sites were identified, grouped into three categories: main routes (0–100 cw-km), secondary corridors (100–5,000), and wider but more costly routes (>5,000). Truncation to 10,000 cw-km allowed the isolation of networks with increased ecological relevance. Circuitscape complemented this analysis by identifying areas of high cumulative flow (cumcurmap), territorial influence (cwd), and individual connectivity potential (res_voltmap).

The comparative analysis demonstrated significant spatial convergence between the two models, especially in common mobility corridors (valleys, passes, low plateaus between 300–1200 m altitude). Some sites (Ungureasca Cave, Peștereanu Cave, Curată Cave) show a high degree of centrality in the network, while others, such as the caves in Vârghiș Gorges, appear isolated or poorly connected to the rest of the area, but have close links between them. The correlation between the model and the topographical reality allowed the validation of the hypotheses regarding the preferential routes and the exclusion of areas with prohibitive costs.

**Fig 11:**
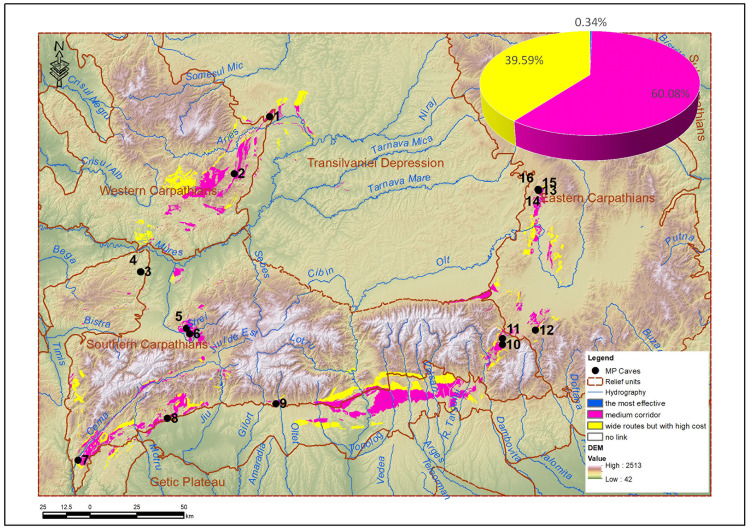
Karst areas in the Romanian Carpathians intersected by mobility corridors.

The intersection of the mobility corridors with the map of the karstic relief [43] in the analyzed area results in a space that is suitable for future analyses, for future research and for the identification of new possible sites (Fig 11).

Fig 11 presents the result of intersecting the modeled mobility corridors with the karst relief areas, in order to identify those spaces where plausible prehistoric mobility overlaps with geological substrates favorable for the conservation of archaeological sites. This figure synthesizes a spatial analysis with high applicative value and provides a prospective framework for directing field research.

The karst map used in the analysis was derived from the Geological Map of Romania [56], by extracting and vectorizing the surfaces covered by karstifiable rocks (limestone, dolomite, travertine), considered relevant for the presence of caves or natural shelters.

The intersection of these karst units with the network of energy corridors generated with Linkage Mapper led to the definition of a potential archaeological research space, where accessibility conditions and geomorphological potential combine. The resulting areas were classified according to the level of energy cost:

- 7.9 km² are associated with the corridors with minimum cost (maximum energy efficiency),
- 1416.3 km² are found in intermediate corridors, with moderate costs,
- 933.2 km² belong to peripheral areas, with high costs, but included in the network.

In total, the 2357 km² resulting (representing approximately 2.6% of the total area analyzed) can be interpreted as areas of priority interest for future archaeological prospecting, especially in poorly researched karst regions.

This analysis not only supports the interpretation of Neanderthal mobility, but also provides a practical tool for selecting target areas, based on a combination of functional movement logic and favorable geological substratum.

Finally, the proposed methodology provides a replicable framework for investigating Paleolithic mobility and allows prioritizing future archaeological investigations based on spatial probability of connection. The study proves the added value of GIS modeling in archaeological analysis and supports the integration of these methods in prehistory studies at regional and European scales.
